# The missing thread of One Health efforts: improper drug disposal as an overlooked driver of antimicrobial resistance

**DOI:** 10.1128/msphere.00586-25

**Published:** 2026-04-13

**Authors:** Sandip Mukhopadhyay, Falguni Debnath, Debjit Chakraborty

**Affiliations:** 1 ICMR-National Institute for Research in Bacterial Infections 30170 , Kolkata, India; 2 Model Rural Health Research Unit-Darjeeling, Darjeeling, West Bengal, India; University of Nebraska Medical Center College of Medicine, Omaha, Nebraska, USA

**Keywords:** drug disposal, antimicrobial resistance, take-back service, resistome, ecopharmacovigilance, environmental monitoring

## Abstract

With gradual recognition of the components and the stakeholders, “One Health approach” became a global strategy for mitigating antimicrobial resistance (AMR). However, the role of improper pharmaceutical disposal, particularly antimicrobials at the household level, remains largely overlooked within One Health strategies. Expired and unused medicines are frequently discarded into household waste, drains, or open environments. The bioactive pharmaceutical residues enter soil, surface water, groundwater, and sediments. Conventional waste management and wastewater treatment systems are not designed to remove these compounds, resulting in chronic, low-level environmental exposure. Such sub-inhibitory concentrations of antimicrobials exert sustained selective pressure on environmental microbial communities, which promotes the emergence, persistence, and dissemination of resistant bacteria. Discarded antimicrobials persist in aquatic and terrestrial ecosystems, reshape microbial communities, disrupt nutrient cycling, and accelerate horizontal gene transfer. The environmental resistome, a vast genetic reservoir connecting environmental microbes with human and animal pathogens, plays a key role in resistance amplification. Evidence from India and other low and middle-income countries reveals the widespread presence of “clinically important resistance genes,” including extended-spectrum β-lactamases and carbapenemases, in non-clinical environments. Residues and resistant bacteria can bioaccumulate in aquatic organisms and livestock, facilitating transmission through food chains and communities and often beyond routine surveillance. Despite its significance, household pharmaceutical waste management is largely absent from national and global AMR action plans. Incorporating safe drug disposal may serve as the missing thread in the One Health, apart from environmental monitoring and ecopharmacovigilance, which are critical to reduce environmental selection pressure and resistance propagation.

## INTRODUCTION

Improper disposal of expired or unused medicines from the households may have consequences extending far beyond household boundaries. Although pharmaceutical industries often maintain organized systems to retrieve expired products from hospitals and pharmacies, there is no access to such a structured “take-back system” for millions of families worldwide (1, 2). People often routinely throw tablets and capsules into household trash, flush them down toilets, or pour them into drains (3). The lack of accessible disposal facilities, coupled with limited awareness of safe disposal practices, fosters a widespread culture of unsafe medication disposal (4). The conventional solid bio-waste management system is not designed to capture or degrade pharmaceutical chemicals. Hence, once discarded, these compounds can leach into soil, seep into groundwater, and contaminate lakes, ponds, and rivers, leading to bioaccumulation in aquatic life (5). Over time, residues from improperly managed solid waste enter local waterbodies and disrupt the aquatic ecosystems (6).

It is not just the poorly managed solid wastes, the domestic wastewater, livestock effluent, landfill leachate, and the “agricultural runoff”—all contribute to the pharmaceutical burden in natural waters. Most wastewater treatment plants lack the advanced processes needed to remove or degrade these compounds effectively (7, 8). As a result, the drug residues persist and recirculate through interconnected environmental compartments—fields, irrigation channels, ponds, and rivers. Their presence has been documented even in drinking water sources (9). Despite being a cause of concern, household pharmaceutical waste remained an environmental and public health “blind spot.” The absence of clear guidelines, minimal community-based collection systems, and poorly sustained awareness efforts reflects a notable global policy gap. Humans, through daily interactions with contaminated water and food, remain indirectly exposed to health hazards arising from these contaminated environments (8, 10).

**Fig 1:**
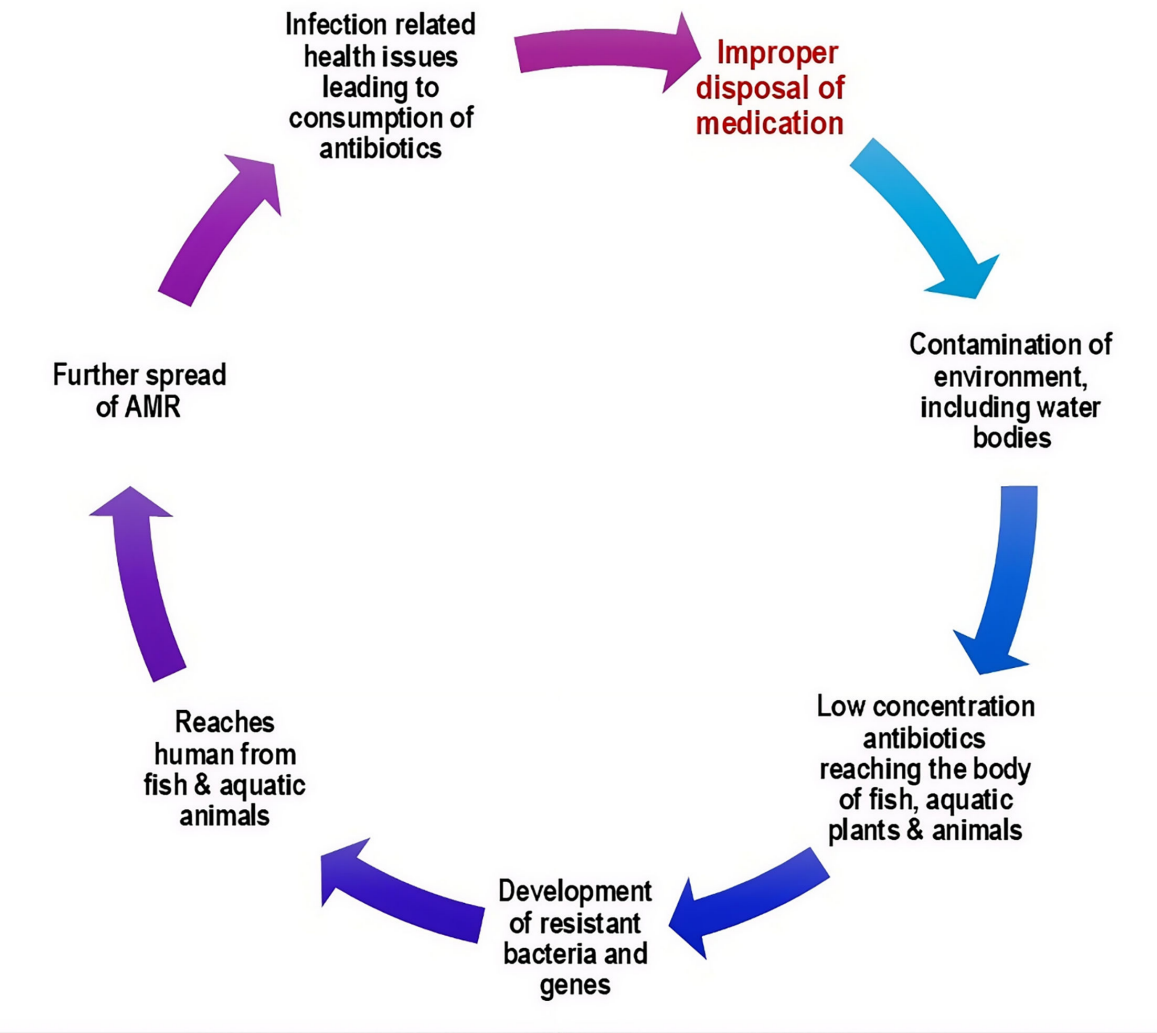
The vicious cycle of improper drug disposal to drug resistance.

When antibiotics or other antimicrobials form part of this mismanaged waste, the consequences are even more concerning (11). Aquatic environments often contain low, sub-inhibitory concentrations of antibiotics that exert selective pressure on naturally occurring microbial populations. Continuous low-level antibiotic exposure fosters the selection and proliferation of resistant bacterial strains (12). This facilitates the horizontal gene transfer (HGT) phenomenon, where the resistance genes spread among bacterial populations in soil and water (13). The act of “waste disposal” slowly becomes a promoter of antimicrobial resistance (AMR). The resistant bacteria and resistance genes migrate through aquatic animals and community water sources, eventually to the human food chain. Such microbial changes occur silently and remain undetectable unless subjected to methodological surveillance (14, 15). Resistant strains emerging from such neglected environmental niches can disperse across communities and borders, transforming local inattention into a broader, transboundary health challenge. Hence, improper domestic antimicrobial disposal has the potential to generate a vicious cycle leading to the emergence of AMR and spreading it further. These interconnected processes are summarized in Fig. 1.

Despite being a significant environmental and public health concern, improper antibiotic disposal still receives minimal attention in global AMR strategies (16). Current One Health frameworks heavily emphasize antimicrobial use in humans, animals, and agriculture, but often fail to address the environmental life cycle of discarded medicines. This oversight leaves a critical gap in AMR prevention. Recognizing improper drug disposal as an environmental hazard and a contributor to AMR is essential for building a more complete One Health response (17, 18).

The current review focuses on the neglected link between improper drug disposal and antimicrobial resistance, exploring environmental pathways, public health implications, and policy solutions for an integrated One Health approach.

## CURRENT SCENARIO OF DRUG DISPOSAL PRACTICES

Unused and expired medicines are accumulating globally. The gap between policy and practice is especially wide in low and middle-income countries (LMICs), where environmental regulations, pharmaceutical stewardship, and public awareness are still evolving. Disposal of antimicrobials and surplus pharmaceuticals at the household trash bins was found to be the most common way of disposal in African countries like Ghana, Zambia, or Nigeria, or even in Asia like Afghanistan or Nepal (19–22). Countries with the largest populations are obviously large consumers of pharmaceutical products. However, a populous country like India is yet to develop a national-level structured take-back program from the households, though their hospitals often segregate biomedical waste. As evidenced in multiple surveys across Indian states, 70%–90% of unused medicines are disposed of in household waste or flushed (23). It is expected that a proportion of these pharmaceutical products are antimicrobials, and when thrown in household garbage, flushed, or burned, they contaminate soil and water, disrupt ecosystems, and contribute to AMR. Large OTC consumption and self-medication practices in India may further aggravate the scenario. On the positive side, some major pharmacy stores now informally offer collection bins or take-back service for unused medications in Indian cities like Bengaluru, Hyderabad, Mumbai, and Delhi, and a program like nPROUD (New Programme for Removal of Unused Drugs) has been initiated in Kerala, an Indian state (24). Environmental AMR surveillance is also being taken more readily by the Indian research bodies. Compared to most LMICs, China has a stronger environmental enforcement and has municipal take-back bins in many cities (Shanghai, Beijing, and Guangzhou), often in collaboration with community pharmacies. However, the improper disposal is still high in many parts, including the rural areas, reflecting a fragmented ecosystem of drug disposal (25). China shows high levels of antibiotic pollution in certain regions, which is also likely in India due to dense manufacturing clusters (26).

The Western world, including the USA, the European Union, and the UK, provides more structured, accessible take-back systems, supported by legislation and public awareness. Despite that, a systematic review reported that, like Kuwait, Qatar, Ghana, Bangladesh, or Saudi Arabia, the most common method for disposal of unused medications in households is disposal in the garbage, even in the United Kingdom or Lithuania. Lack of adequate information and clear instructions on proper manners of drug disposal was noted even in the developed nations like the United States, New Zealand, and Ireland, similar to Bangladesh (27). Hence, household-level drug disposal is a matter of concern for both the LMICs and many of the developed countries with smaller populations. Damage to the ecosystem and vulnerability should therefore be considered as a global phenomenon rather than a country-centric problem. These heterogeneous and often unsafe disposal practices create multiple environmental entry points for antimicrobials, which are detailed in the following sections

## ANTIMICROBIALS: ENVIRONMENTAL PATHWAYS AND MICROBIAL CONSEQUENCES

Antimicrobials have been detected in different environments—ranging from groundwater, lakes, surface water, river water, sediments, sewage, soils to the ocean (28). Whatever may be the route of release, from domestic effluent, pharmaceutical, agricultural fields, or animals, once released into the environment, antimicrobial compounds undergo dilution but remain biologically active even at sub-inhibitory concentrations. These residual antibiotics create chronic low-level selective pressure on environmental bacteria. Gullberg et al. experimentally proved that very low concentration of tetracycline, ciprofloxacin, and streptomycin, in a concentration similar to that found in some aquatic and soil environments, was associated with the development of resistant strains of *Escherichia coli* and *Salmonella enterica* (var. Typhimurium LT2). The authors observed “rapid enrichment of *de novo* resistant mutants (29).” Over time, such low-level exposure drives the evolution and persistence of resistant strains, contributing to the environmental resistome—a vast pool of resistance genes in nature (30, 31).

Differences in microbial communities between antibiotic-exposed and unexposed soil and water systems were clearly evident in some controlled studies. Under experimental conditions, very low antibiotic concentrations resulted in enrichment for streptomycin, tetracycline, and ciprofloxacin-resistant mutants of *Escherichia coli* and *Salmonella enterica* (29). It was found that long-term antibiotic exposure in soil alters bacterial community structure and increases antimicrobial resistance genes (ARGs) (32). Similarly, in aquatic environments, antibiotic release changes bacterial dynamics and reduces denitrifier activity in sediments and groundwater. A recent microcosm study showed that antibiotic exposure significantly altered prokaryotic diversity while fungal communities remained stable. These findings underscore the harmful long-term ecological impacts (33, 34).

Antibiotics from improperly disposed drugs can enter

- Aquatic systems, through leaching or direct dumping, contaminating rivers, ponds, and groundwater;
- Soil ecosystems, where they influence microbial composition and reduce biodiversity;
- Food chains, via accumulation in aquatic plants, fish, and animals, eventually reaching humans.

Drinking water is derived from surface water. Hence, water serves as a medium for the dissemination of “antibiotic-resistant organisms” to humans or even animals. Introduction of the resistance genes to natural bacterial ecosystems also follows a similar route (30). Aquatic system and the sub-inhibitory exposure not only promotes resistance but also facilitates horizontal gene transfer among commensal and pathogenic bacteria (35). These environmental reservoirs act as silent incubators for AMR, from which resistant bacteria can return to human populations via contaminated food or water.

Improper disposal of antimicrobials allows bioactive compounds to infiltrate the environment, where they continue to exert selective pressure on microbial populations. The environmental fate of antibiotics and their metabolites is determined by multiple interconnected pathways—soil, water, sediment, and living organisms—all of which serve as reservoirs and conduits for AMR proliferation. Unlike clinical or agricultural misuse, this environmental exposure is unregulated, chronic, and diffuse, making it both difficult to monitor and nearly impossible to reverse.

The major pathways and mechanisms by which improperly discarded antimicrobials influence environmental microbiota and foster resistance evolution are described in the following sections.

### Household disposal as the entry of antimicrobials into the environment

Improper drug disposal—through flushing, open dumping, or mixing with household waste—may serve as the initial entry point of antimicrobials into the environment. Expired and unused medicines are often discarded into municipal bins, ponds, or drainage channels. During monsoon runoff, these substances mix with surface water and percolate into the soil and groundwater (36).

Several studies have reported detectable concentrations of antibiotics such as ciprofloxacin, amoxicillin, azithromycin, and sulfamethoxazole in surface and groundwater near waste disposal sites (28, 37). These residues persist due to their chemical stability and resistance to biodegradation. Pharmaceuticals are designed to withstand metabolic breakdown within the body; consequently, when released into the environment, they persist long enough to influence microbial ecosystems (38).

Even wastewater treatment plants, where they exist, are not specifically designed to remove antibiotics. Hence, effluents discharged into rivers or reused for irrigation continue to carry trace quantities of antimicrobials, extending exposure to soil and aquatic microorganisms (39, 40).

### Fate of antimicrobials in soil ecosystems

Soil acts as both a sink and a source of antibiotic residues. When discarded drugs enter soil through leaching or dumping, they adsorb onto clay particles and organic matter, forming stable complexes. The bioavailability of these compounds depends on soil pH, texture, and microbial activity (41).

Within this microenvironment, sub-inhibitory concentrations of antibiotics alter microbial community structure and function. Commensal soil bacteria exposed to these agents experience selective pressure that favors the survival of resistant mutants. Even non-pathogenic soil bacteria can develop or acquire resistance genes under such pressure, turning soil into a vast reservoir of resistance determinants (32).

Studies have identified ARGs such as *blaTEM*, *tetA*, and *sul1* in agricultural soils receiving wastewater irrigation or sludge. These genes can persist long after the parent antibiotic degrades, creating a genetic legacy of resistance. Furthermore, resistant soil bacteria may transfer their genes to plant-associated microbes or enteric pathogens through HGT (42, 43).

Soil contamination is not a static process. Through dust, runoff, or crop uptake, these resistant organisms and genes can spread beyond the disposal site, integrating into food webs and human-associated environments (44).

### Aquatic pathways, persistence, and biofilms

Water bodies represent the most dynamic and significant reservoirs for antibiotic contamination. When households, healthcare facilities, or waste collectors dump drugs into drains or rivers, the compounds dissolve and distribute across large water volumes, creating low but continuous antibiotic exposure for aquatic microorganisms (45).

Pharmaceutical residues have been detected in nearly all environmental water matrices—from sewage and hospital effluents to rivers and coastal waters (40). Even nanogram-per-liter concentrations are sufficient to exert evolutionary pressure on bacteria. For instance, *E. coli* and *Pseudomonas aeruginosa* can develop fluoroquinolone resistance when exposed to ciprofloxacin concentrations as low as 0.1 µg/L over several generations (46, 47).

These aquatic environments often harbor complex microbial communities where selective pressure favors the maintenance of plasmids carrying resistance genes. Biofilms may be found attached to any submerged surface or solid-liquid interface, such as stones, wood, shells, sediment, or on other living aquatic organisms and often contain microcolonies of bacterial cells within an extracellular polymeric substance matrix (48). In biofilms formed on aquatic sediments and surfaces, bacterial density and proximity facilitate gene exchange via conjugation or transformation (49). Resistant genes that emerge here can be mobilized into clinical pathogens when water is used for irrigation, drinking, or aquaculture.

Furthermore, antibiotic residues in rivers may reach estuarine and marine ecosystems, affecting phytoplankton, zooplankton, and fish microbiota (50). Aquatic species thus become carriers of resistant bacteria, facilitating their movement through food webs and trade routes.

### Sediment and biofilm reservoirs

Sediments serve as long-term repositories for antibiotic residues. Compounds like tetracyclines and fluoroquinolones accumulate in riverbeds and ponds. On the other hand, biofilms may form on sediment particles leading to a stable, cohesive layer altering sediment transport, nutrient cycling, and pollutant trapping including antimicrobials. Gene exchange and survival under stress become easier for bacteria present within biofilms in the sediments due to the networks and proximity of the cells in biofilms (49, 51). Exposure of the bacteria to the antimicrobials in such a “supportive environment” promotes antimicrobial resistance, making the sediments as sources for virulent microorganisms and biofilms as “hot spots of antibiotic resistance” (52). Studies from the sediments of the Indian rivers reveal high densities of multidrug-resistant bacteria and resistance genes, including blaNDM-1 (53). Biofilms act as persistent reservoirs that can release resistant bacteria during floods or dredging.

### Impact on aquatic and terrestrial biota

Once the aquatic environment gets contaminated by the antibiotic residues and resistant bacteria, the impact spreads beyond the microorganisms. Fish and aquatic plants bioaccumulate antimicrobials. Even at low concentrations, these residues disrupt normal gut microbiota, impair immune responses, and are associated with reduced growth, altered metabolism, and increased susceptibility to disease (54). The effect is not limited to ponds or lakes; even the marine fishes and animals were not exempted. Sulfonamides, trimethoprim, and fluoroquinolones were found to accumulate primarily in the muscles and macrolides in the liver of the wild marine fishes (55). Cultured fishes, on the other hand, are often exposed to antibiotic use as an aquaculture practice.

Importantly, fish can become carriers of antimicrobial resistance. Studies have reported the presence of extended-spectrum β-lactamase-producing *Escherichia coli* and *Enterobacter* species in fish harvested from polluted rivers (53). Alarmingly, even in the developed countries with wastewater treatment, river fish was detected to harbor antimicrobial-resistant bacteria, antimicrobial resistance genes (ARGs), and bioaccumulated antibiotic residue (56). These resistant bacteria may persist in fish intestines and on gill surfaces, facilitating their entry into food chains.

In terrestrial ecosystems, livestock drinking contaminated water or feeding on land irrigated with polluted water or vegetation grown in antibiotic-laden soil may acquire resistant bacteria in their gut flora. These organisms can then spread to humans through the consumption of inadequately cooked fish, direct animal contact, handling of raw animal products, or consumption of meat. In this way, contaminated water environments form a critical but often overlooked bridge linking environmental antibiotic pollution with animal reservoirs and human infections, reinforcing the One Health continuum of antimicrobial resistance (57).

### Mechanisms of resistance selection and gene transfer

The emergence and persistence of resistance in environmental bacteria are primarily driven by sub-inhibitory antibiotic concentrations, a level too low to kill bacteria but high enough to induce stress responses (58). This triggers the SOS response, mutagenesis, and activation of efflux pumps, all contributing to the evolution of resistant phenotypes of the bacteria (59).

Additionally, antibiotic exposure enhances HGT, a major driver of AMR dissemination. HGT occurs through (i) conjugation (direct plasmid transfer between bacterial cells), (ii) transformation (uptake of free DNA from lysed cells), or through (iii) transduction (bacteriophage-mediated gene movement). Environmental conditions contaminated with antibiotics increase bacterial density, stress responses, and biofilm formation—factors that collectively favor these gene-sharing processes (60).

Consequently, resistance genes spread rapidly across multiple species, connecting environmental bacteria with clinically relevant pathogens. Mobile genetic elements such as plasmids, integrons, and transposons serve as vehicles, allowing resistance traits to move between environmental bacteria and human pathogens (61).

### Antibiotic residues as environmental pollutants

Pharmaceutical residues are emerging contaminants that continuously influence microbial evolution at trace levels. They reduce microbial diversity and disturb nutrient cycling (62). Moreover, antibiotic residues often coexist with other pollutants such as heavy metals, disinfectants, and pesticides. This coexistence creates powerful “co-selection pressures,” where resistance to one stressor inadvertently promotes resistance to others. Genes conferring metal tolerance or biocide resistance are frequently linked on the same plasmids or integrons as antibiotic resistance genes. This enables cross-resistance and co-resistance even in the absence of direct antibiotic exposure. In such chemically complex environments, resistant bacteria gain a competitive advantage, allowing antimicrobial resistance to persist and spread long after antibiotic concentrations decline. These synergistic interactions help explain why AMR is remarkably resilient in mixed-contaminant settings, such as municipal waste streams, hospital effluents, and urban rivers (63).

### The environmental resistome: a hidden threat

The environmental resistome is the vast collection of antibiotic resistance genes harbored by environmental microorganisms. This encompasses both harmless and pathogenic microorganisms that inhabit soil, water, sediments, and wastewater. It acts as a genetic reservoir that can supply new resistance determinants to human pathogens. While most natural bacteria have intrinsic resistance mechanisms, human activities like antibiotic pollution, untreated wastewater discharge, and agricultural runoff greatly amplify the abundance, persistence, and mobility of these genes (57, 64).

Environmental monitoring in India has detected clinically relevant ARGs—such as *blaCTX-M*, *mecA*, and *qnrS*—in river and sewage samples. The presence of these genes outside clinical settings signifies environmental amplification. Once resistance determinants are established in environmental niches, eradication becomes practically impossible (51, 53).

This resistome represents a continuum between environmental, animal, and human health. It indicates that the One Health perspective that AMR should not be confined to hospitals as the resistome thrives in the interconnected biosphere (65).

## PUBLIC HEALTH IMPLICATIONS

The health risks posed by improper drug disposal extend beyond environmental contamination. Resistant bacteria and resistance genes originating from aquatic and soil ecosystems can spread to humans through direct contact, agricultural use, or food consumption. With rampant travel and migration, this silent cycle makes AMR a transboundary issue, as resistant strains can cross national borders through migratory birds, trade, and shared water systems (66).

The risk is particularly pronounced in geographically sensitive regions such as India’s “chicken’s neck corridor,” which borders Nepal, Bangladesh, and Bhutan. This narrow tract of land facilitates human and material movement across countries, creating an ecological and epidemiological hotspot for AMR spread. Studies have detected resistant *Enterobacteriaceae*, *Klebsiella*, and *Pseudomonas* species in environmental and clinical samples, underscoring the interconnectedness of ecosystems (67). The matter needs special attention as the whole “South East Asia” has a concerning level of AMR (68).

Improper pharmaceutical waste management thus not only threatens local ecology but also undermines regional and global AMR containment efforts.

## POLICY GAPS AND MISSED OPPORTUNITIES

Despite growing recognition of AMR as a One Health issue, improper drug disposal remains practically absent in a vast majority of countries. Large pharmaceutical producer and consumer like India has rolled out “National Action Plan on Antimicrobial Resistance (NAP-AMR) 2.0,” with “One Health approach,” but did not link the strategic objectives explicitly to pharmaceutical waste management (69). Current waste management policies primarily address biomedical and industrial waste but fail to regulate domestic or over-the-counter pharmaceutical residues (70).

Key challenges include the following:

- Absence of community-level drug collection programs or pharmacy take-back schemes;
- Lack of standardized protocols for safe household disposal of pharmaceuticals;
- Poor intersectoral coordination among health, environment, and municipal departments;
- Limited public education and awareness campaigns (71).

In contrast, many high-income countries have established pharmacy-led collection systems (e.g., take-back programs in the USA, return unwanted medicines in Australia), which have effectively reduced environmental exposure by removing surplus medications. Studies report the collection of thousands of pounds annually, though representing <1% of dispensed controlled drugs, with mail-order prescriptions yielding 3× more waste per return. Comparative data show higher ARG levels and resistance rates in soils/waters without such programs versus areas with recycling, linking take-back to lower environmental AMR burdens (33, 72, 73). These initiatives effectively reduce environmental exposure and promote responsible disposal behavior. The LMICs must adapt such models to minimize the critical gap in AMR prevention efforts. Recognition of the environmental pathways of AMR has profound implications for surveillance. Current systems focus on clinical isolates, neglecting environmental sources. Integrating antibiotic residue and resistance gene monitoring in soil and water into national surveillance programs is crucial. Sentinel sites near manufacturing clusters and dumping grounds can serve as early warning systems for emerging resistance.

## RECOMMENDATIONS AND WAY FORWARD

**Fig 2:**
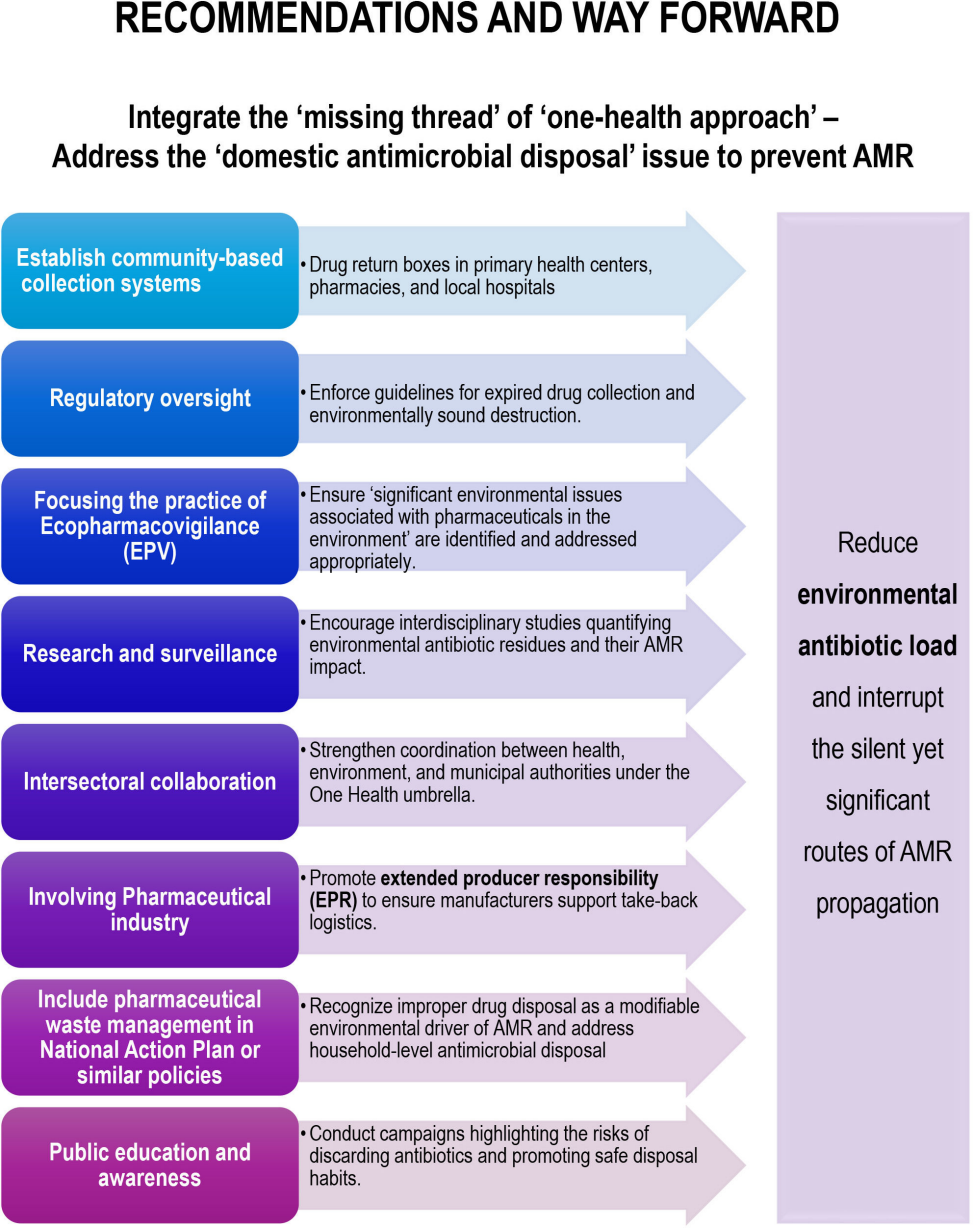
Recommendations and way forward: address the “domestic antimicrobial disposal” issue to prevent AMR.

Addressing the “domestic antimicrobial disposal” issues should link the “missing thread” of “one-health approach” in the prevention of AMR. However, it requires multi-pronged interventions involving communities, health systems, and policymakers to address the issue of improper drug disposal worldwide (Fig. 2). Hence, all the following strategies may be important.

- Establish community-based collection systems: integrate drug return boxes in primary health centers, pharmacies, and local hospitals. Kerala, the first state of India, rolled out nPROUD, which is a government-led drug take-back program, involving household collection drives and pharmacy drop-offs (74). At the same time, some NGO-run projects like “Project Green Pharmacy” came up with similar objectives in other states and improved the awareness and practice of disposal (75). Take-back service or similar safe disposal of pharmaceuticals, including the antimicrobials, must extend to every place of the globe.
- Developing drug waste recycling: implementing drug waste recycling requires pharmacy collection bins, secure transport logistics, and centralized incineration/high-temperature facilities to destroy residues safely. Common strategies include physical segregation/storage in sealed bins and chemical incineration (>1,000°C) to prevent leaching (71, 76, 77). Cost estimates vary: the initial setup for small programs is approximately $5,000–$20,000 (bins, training), with annual operating costs of $10,000–$50,000 for LMICs via shared models (e.g., India's nPROUD with govt/pharmacy funding) compared with $4.12 million to $9.62 million in other places (78, 79). Cement kiln co-processing provides an energy-efficient alternative capable of degrading pharmaceutical residues. For selected hazardous drugs, encapsulation or inertization may be appropriate. At the wastewater treatment level, advanced tertiary processes such as ozonation, activated carbon adsorption, and advanced oxidation technologies enhance removal of residual antibiotics that escape primary treatment. However, economic feasibility remains a challenge, particularly in low and middle-income countries. Hence, phased district-level implementation and extended producer responsibility (EPR) models, where manufacturers share logistical costs, can substantially reduce financial barriers (80–82).
- Regulatory oversight: it is important to develop guidelines as well as enforce the guidelines for disposal or collection of expired drugs, including the antimicrobials, and arrange destruction in an environmentally sound way.
- Focusing on the practice of ecopharmacovigilance (EPV): pharmacovigilance, which ensures the safety of pharmaceuticals for human health, had been popularized beyond the first world. EPV may be considered as its counterpart with a focus on the environment, aiming to ensure that “significant environmental issues associated with pharmaceuticals in the environment” are identified and addressed appropriately (83). Undoubtedly, this area needs more awareness with possible integration in the policies.
- Research and surveillance: encourage interdisciplinary studies quantifying environmental antibiotic residues and their AMR impact.
- Intersectoral collaboration: strengthen coordination between health, environment, and municipal authorities under the One Health umbrella.
- Involving the pharmaceutical industry: promote EPR to ensure manufacturers support take-back logistics.
- Include pharmaceutical waste management in the National Action Plan or similar policies globally: such plans should recognize improper drug disposal as a “modifiable environmental driver of AMR” and include household-level antimicrobial disposal in a proper way. A program like NAP-AMR in India is a timely step.
- Public education and awareness: conduct campaigns highlighting the risks of discarding antibiotics and promoting safe disposal habits.

**Fig 3:**
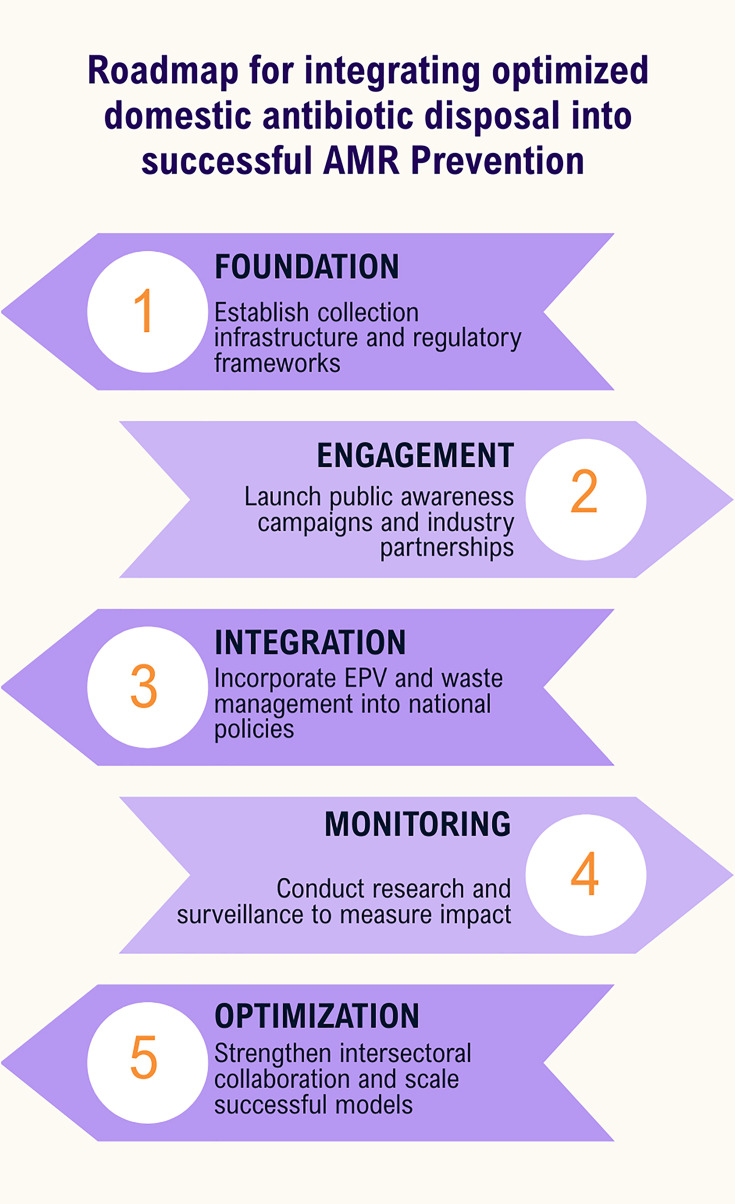
Roadmap for integrating domestic antimicrobial disposal to One Health framework.

Implementing these measures can reduce environmental antibiotic load and interrupt one of the silent but significant routes of AMR propagation. A roadmap is necessary to achieve this goal step by step (Fig. 3).

## CONCLUSION

Improper disposal of pharmaceuticals, especially antimicrobials, constitutes an invisible yet potent contributor to antimicrobial resistance. Many countries globally are still lacking a systematic drug take-back mechanism and handle pharmaceutical waste poorly. The resulting selective pressure nurtures resistant microorganisms that traverse ecological and national boundaries.

It is clear from the existing body of evidence that improper disposal of antimicrobials or pharmaceutical wastes is the unrecognized area in the One Health framework. Recognizing pharmaceutical waste management as an essential component of AMR control is imperative. A coordinated approach—linking health, environment, and policy sectors within the One Health framework—is essential to mitigate this neglected pathway of resistance transmission. Ecopharmacovigilance must be strengthened everywhere, along with the spread of awareness on handling and disposal of antimicrobials, along with other pharmaceuticals.

Immediate integration of safe drug disposal strategies into AMR containment plans globally will not only curb environmental contamination but also protect future generations from the escalating threat of untreatable infections.
